# Diversification of methanogens into hyperalkaline serpentinizing environments through adaptations to minimize oxidant limitation

**DOI:** 10.1038/s41396-020-00838-1

**Published:** 2020-11-30

**Authors:** Elizabeth M. Fones, Daniel R. Colman, Emily A. Kraus, Ramunas Stepanauskas, Alexis S. Templeton, John R. Spear, Eric S. Boyd

**Affiliations:** 1 Department of Microbiology and Immunology, Montana State University, Bozeman, MT 59717 USA; 2 Department of Civil and Environmental Engineering, Colorado School of Mines, Golden, CO 80401 USA; 3 Bigelow Laboratory for Ocean Sciences, East Boothbay, ME 04544 USA; 4 Department of Geological Sciences, University of Colorado, Boulder, CO 80309 USA

**Keywords:** Ecology, Evolution

## Abstract

Metagenome assembled genomes (MAGs) and single amplified genomes (SAGs) affiliated with two distinct *Methanobacterium* lineages were recovered from subsurface fracture waters of the Samail Ophiolite, Sultanate of Oman. Lineage Type I was abundant in waters with circumneutral pH, whereas lineage Type II was abundant in hydrogen rich, hyperalkaline waters. Type I encoded proteins to couple hydrogen oxidation to CO_2_ reduction, typical of hydrogenotrophic methanogens. Surprisingly, Type II, which branched from the Type I lineage, lacked homologs of two key oxidative [NiFe]-hydrogenases. These functions were presumably replaced by formate dehydrogenases that oxidize formate to yield reductant and cytoplasmic CO_2_ via a pathway that was unique among characterized Methanobacteria, allowing cells to overcome CO_2_/oxidant limitation in high pH waters. This prediction was supported by microcosm-based radiotracer experiments that showed significant biological methane generation from formate, but not bicarbonate, in waters where the Type II lineage was detected in highest relative abundance. Phylogenetic analyses and variability in gene content suggested that recent and ongoing diversification of the Type II lineage was enabled by gene transfer, loss, and transposition. These data indicate that selection imposed by CO_2_/oxidant availability drove recent methanogen diversification into hyperalkaline waters that are heavily impacted by serpentinization.

## Introduction

Hydrogen (H_2_) links the geosphere to the biosphere and has likely done so since early in Earth’s history [1–4]. H_2_ can be generated through hydration and oxidation of ferromagnesian minerals (e.g., olivine) in mafic and ultramafic rocks during serpentinization [5], a geochemical process that has been proposed to have supported life on early Earth [6, 7]. High concentrations of H_2_, in turn, can abiotically reduce dissolved inorganic carbon (DIC) to generate reduced carbon substrates including carbon monoxide (CO), formate (HCOO^−^), and methane (CH_4_) [8, 9], additional potent reductants capable of fueling microbial metabolism [10–19]. However, the process of serpentinization also generates hyperalkaline waters that are highly reduced and limited in DIC and other oxidants capable of supporting autotrophs, including methanogens and acetogens [19–21]. As such, the process of serpentinization can create competing benefits (replete reductant supply) and challenges (limited DIC and oxidant supply) for autotrophic subsurface microbial life, both today and early in Earth’s history.

Microbial communities that inhabit hyperalkaline waters in serpentinizing environments tend to be less diverse and less abundant than those inhabiting waters with circumneutral pH [10, 18, 19, 22–24], consistent with the polyextremophilic and DIC/oxidant limited conditions commonly found at pH > 10. More specifically, the decrease in diversity among communities that inhabit hyperalkaline waters tends to be associated with a shift toward a predominance of strict anaerobes, a finding that is in line with the highly reduced nature of these fluids [19, 22]. Among the anaerobes commonly detected in hyperalkaline serpentinized waters are methanogens affiliated with the autotrophic and hydrogenotrophic genus, *Methanobacterium* [13, 14, 18, 19, 23, 25].

Hydrogenotrophic methanogens are often advocated as having the most ancient of extant metabolisms [4, 26, 27]. There are numerous rationales for the primacy of hydrogenotrophic methanogens. First, CH_4_ in fluid inclusions in ancient rocks dated to 3.46 Ga are isotopically light, consistent with active methanogenesis at this time [28], and phylogenetic data suggest that these cells were likely dependent on H_2_ as the electron donor [2, 4]. Second, the ability of hydrogenotrophic methanogens to use CO_2_ as the sole carbon source and electron acceptor may have alleviated a major barrier for early autotrophic life. CO_2_ is presumed to have been readily available on early Earth whereas organic carbon and other potential oxidants (e.g., oxygen, nitrate, and sulfate) are likely to have been far more limiting [29]. However, modern serpentinizing environments exhibit extremely low concentrations of DIC [13, 14, 18, 19, 23, 25], and it is not yet understood how methanogens or other autotrophs could have overcome this limitation.

CH_4_ in hyperalkaline, highly serpentinized waters typically exhibits isotopically heavy carbon (δ^13^C-CH_4_>−40‰) [13, 25, 30, 31], a finding that has been suggested to result from abiotic processes or biological methanogenesis under extreme DIC limitation [25, 32, 33]. Support for the latter interpretation includes experimental evidence demonstrating that biological methanogenesis under carbon-limited conditions can result in relatively high δ^13^C-CH_4_ values [34] and the presence of lipid biomarkers associated with methanogenic archaea recovered from globally distributed serpentinizing environments that exhibit high ^13^C values [35, 36]. These observations point to near-complete (quantitative) consumption of bioavailable DIC by methanogens in these environments. Furthermore, cells in hyperalkaline waters in serpentinizing systems tend to preferentially assimilate carbon substrates for biomass synthesis rather than dissimilate them for energy production when compared to cells from waters with circumneutral pH [18]. This is consistent with cells, including autotrophic methanogens, experiencing DIC limitation in hyperalkaline waters [18, 37]. Here, we apply metagenomics and single cell genomics (SCG) to examine how autotrophic methanogens overcome DIC/oxidant limitation imposed by highly reducing, high pH conditions in the Samail Ophiolite, Sultanate of Oman, the largest ophiolite and one that is undergoing active low temperature serpentinization [20, 25, 38–42]. Microcosm assays utilizing radioisotopically labeled substrates are used to evaluate hypotheses generated from metabolic reconstructions inferred from genomic data. Finally, phylogenomic and SCG approaches are used to describe the diversification of methanogens in hyperalkaline waters resulting from serpentinization and to identify the mechanisms involved in their apparent speciation.

## Materials and methods

### Sample collection

A Grundfos SQ 2–85 submersible pump was used to collect water samples in February 2017 from seven previously drilled wells in the Samail Ophiolite, Sultanate of Oman, as previously described [18, 19]. Briefly, waters were collected at least 20 m below the water table in each well, including two depths at well NSHQ14: 50 m (NSHQ14B) and 85 m (NSHQ14C). After pumping ~100 L of water through the tubing and filter housing, biomass was collected using in-line 0.2 µm Millipore polycarbonate filters in 47 mm Pall polycarbonate filter housings, as described previously [18]. Filters and their contents were transferred to sterile screw top vials and were immediately flash frozen in the field with liquid nitrogen. Water samples for single cell genomics (SCG) were collected from NSHQ14C only. Filter-sterilized (0.2 μm) glycerol (5% final concentration) and TE buffer (1×) were added to samples as cryoprotectants, after which the samples were flash frozen in liquid nitrogen and stored at −80 °C until further analysis.

A Grundfos SQ 2–85 submersible pump was again used to collect water samples for methanogenic activity assays from well WAB188 at a depth of 50 m on March 6, 2020. A Double Packer Standard System (SolExperts, France) was deployed in well NSHQ14 to attempt to isolate shallow versus deep waters in the well. However, attempts to isolate deep waters were unsuccessful as there was insufficient flow to pump water to the surface when the top packer was inflated (at both 30 m and at 18 m depths). Sufficient flow was achieved when the bottom packer was inflated at a depth of 30 m with the top packer left uninflated, isolating waters from an interval extending between the depth of the water table (9 m) to 30 m on February 28, 2020. After pumping ~100 L of water through the sampling manifold and tubing at each site, water samples for methanogenic activity assays were collected by direct injection from the sampling manifold into N_2_-purged, autoclave-sterilized, butyl-stoppered glass serum vials. Samples were maintained at ambient temperature during transport to the laboratory and were then placed at 4 °C for storage. Water temperature and pH were measured in the field in 2020 with a Hach (Loveland, CO) HQ40D Portable Multi Meter.

### Site description

The classification scheme used here to describe major water types (hyperalkaline peridotite, contact, and alkaline peridotite) in the Samail Ophiolite is as reported previously [18, 19]. Increasing pH is generally interpreted to reflect the extent of serpentinization reaction progress and the degree of fluid mixing in each well [19]. DIC was replete (3.0–3.5 mM) and H_2_ was limited (undetectable to 0.92 μM) in circumneutral waters (pH 7.6–8.5) from wells WAB188, WAB105, and WAB104 at the time of sampling in 2017 (Table 1) [18]. In contrast, DIC was limited (0.05–0.13 mM) and H_2_ was replete (21 to 164 μM) in hyperalkaline waters (pH 11.1–11.3) from well NSHQ14 in 2017. Formate (HCOO^−^), another potential reductant generated during serpentinization, has been measured at concentrations ranging from 1.0 to 1.7 μM in these wells [19]. Further details of the geochemistry of waters sampled from the Samail Ophiolite are reported elsewhere [18, 19].

**Table 1:** Geochemical measurements for well waters sampled in 2017 and 2020.

Well	WAB188		NSHQ14		
Year	2017	2020	2017	2017	2020
Water table depth (m)	9	8.5	12	12	9
Sampled depth (m)	78	50	50	85	9–30
pH	7.6^a^	7.5	11.1	11.3	11.3
Temperature (°C)	33.0^a^	34.7	34.4	36.3	35.7
H_2_ (μM)	0.92	–	21	164	–
DIC (mM)	3.0	–	0.05	0.13	–
Formate (μM)	1.0^b^	–	1.7^b,c^	1.7^b,c^	–
CH_4_ (μM)	1.69	–	34.6	12.6	–

– Denotes data that are not available from the 2020 field season.

Values measured in 2017 were previously reported in Fones et al. [18].

^a^Data collected from wells in 2016 that were previously reported in Rempfert et al. [19] are displayed since measurements were not collected during the 2017 field season.

^b^Data collected from wells in 2015 that were previously reported in Rempfert et al. [19] are displayed since measurements were not collected during the 2017 field season.

^c^The formate concentration was measured in only one depth interval (20 m) in NSHQ14 in 2015, as previously reported in Rempfert et al. [19].

### DNA extraction and shotgun metagenomic sequencing

DNA was extracted for metagenomic sequencing as previously described [18]. Briefly, DNA was extracted from filtered biomass using a Zymo (Irvine, CA) Research Xpedition Soil/Fecal DNA MiniPrep Extraction kit according to manufacturer instructions. Sequencing libraries were prepared in triplicate using the Nextera XT kit (Illumina Inc., San Diego, CA) and sequenced on the Illumina HiSeq 2500 Rapid Run platform (2 × 250 bp).

### Metagenomic assembly and binning of metagenome assembled genomes (MAGs)

Raw sequence reads were quality filtered, trimmed of adapters, and assembled as described previously [18]. Contigs were binned into draft MAGs based on tetranucleotide frequencies and coverage profiles using the MetaBAT software package (v. 0.26.3) [43]. Contigs with ≥98% nucleotide identity to the *Methanobacterium* Type I MAG from WAB188 were classified as Type I and contigs with ≥98% nucleotide identity to the *Methanobacterium* Type II MAG from NSHQ14C were classified as Type II. The number of reads affiliated with *Methanobacterium* Type I or II MAGs as a percentage of the total reads in each metagenome were used to calculate estimated relative abundances of each population.

### Single cell genomics (SCG)

Individual microbial cells were separated by fluorescence-activated cell sorting, lysed by a combination of freeze-thawing and KOH, and their genomes were amplified by WGA-X and subject to low-coverage sequencing and assembly at the Bigelow Laboratory Single Cell Genomics Center, as described previously [44]. MAG and single amplified genome (SAG) data have been submitted to GenBank under the BioProject number PRJNA631088.

### Phylogenomic analyses

Phylogenomic reconstruction of target MAGs (i.e., *Methanobacterium* spp. described below) recovered from the Samail Ophiolite and representative reference Methanobacteria genomes were performed using a concatenation of 103 aligned, single-copy, phylogenetic marker genes, as previously described [45]. Briefly, all genomes classified within the ‘Methanobacteria’ class were retrieved from the Department of Energy-Integrated Microbial Genome (IMG) and National Center for Biotechnology Information (NCBI) GenBank databases. Marker genes were identified from the genome set using Amphora2 [46], individually aligned using Clustal Omega (v.1.2.4) [47], and concatenated. The concatenated alignment was subject to Maximum Likelihood phylogenetic reconstruction in IQ-TREE (v.1.6.11) [48] after identifying the optimal amino acid substitution model (LG + F + R10) via the Bayesian Information Criterion, as implemented in modelfinder [49].

### Metabolic reconstructions of MAGs and SAGs

Gene prediction and annotation for MAGs and SAGs were performed with Prodigal v.2.6.3 [50] as implemented in Prokka v.1.11 [51] specifying default parameters. Following preliminary predictions and annotations for MAGs, gene functions were further examined using the Kyoto Encyclopedia of Genes and Genomes (KEGG) function database [52] with the KEGG Automatic Annotation Server (KAAS) [53]. Genomes were also queried via BLASTp [54] for specific gene functions, as guided by gene contents of closely related genomes. BLASTp searches were conducted using bait sequences specific for active site subunits for each protein or protein complexes. Positive matches for protein homologs in MAGs and SAGs were considered as those with a BLASTp *E*-value of <1 × 10^−6^, >30% amino acid sequence similarity, and >60% of the length of the query sequence. The complete WAB188, NSHQ14B, and NSHQ14C metagenome assemblies were queried for genes encoding homologs of key proteins initially absent from the MAGs (i.e., H_2_-dependent methenyltetrahydromethanopterin dehydrogenase, Hmd, F_420_-dependent methylenetetrahydromethanopterin dehydrogenase, Mtd, and the formate and formate/nitrate transporters, FdhC, FocA, and YfdC, in both Type I and Type II MAGs; the ion-translocating [NiFe]-hydrogenase complex, Mrp-Mbh, in the Type I MAG; the Group 3 F_420_-reducing [NiFe]-hydrogenase, Frh, and Group 3c bifurcating [NiFe]-hydrogenase, Mvh, in Type II MAGs, described below) via BLASTp. Single amplified genomes affiliated with *Methanobacterium* were also queried for proteins missing from the MAGs via BLASTp. Unbinned contigs containing these homologs were manually assigned to MAGs based on consensus with contig coverage profiles and average GC content, as determined using CheckM [55].

Homologs of [NiFe]-hydrogenase were identified by BLASTp using query sequences for active site subunits. Briefly, candidate [NiFe]-hydrogenase catalytic subunits were compiled and combined with references for specific hydrogenase groups, as defined previously [56]. The combined dataset was aligned with Clustal Omega, evaluated manually for the presence of conserved N- and C-terminal CxxC motifs [57], and subjected to Maximum Likelihood phylogenetic analysis, as described above. The putative directionality and redox partner coupling, as determined initially from phylogenetic analyses above, were further evaluated by comparing amino acid conservation in the L1 and L2 motifs to previously characterized hydrogenases [56]. These functional assignments were further assessed by examining the genes co-localized with the genes encoding hydrogenase catalytic subunits for key partner proteins (e.g., F_420_ binding proteins), as determined with the Conserved Domain Database [58] and BLASTp analysis against the NCBI non-redundant database.

### Comparison of MAGs and SAGs

The average nucleotide identities (ANIs) among SAGs and MAGs were calculated using the MUMmer (v4.0.0beta2) average_nucleotide_identity.py script in python [59]. SAGs (B04, E10, J15, M05, P19, O02, and O05) that were found to have insufficient hits to calculate pairwise ANIs with at least one other genome were removed from this analysis. ANIs among the SAGs and the NSHQ14 MAGs were used to construct a dissimilarity matrix that was translated into a Euclidean distance matrix and used to construct a dendrogram using the hclust function in R specifying the “single linkage” agglomeration method [60].

To evaluate the similarity of proteins encoded among the MAGs and SAGs, the percent identities between each encoded protein homolog were first determined. Homologs were defined as those with ≥30% identity to protein coding genes in the NSHQ14C MAG (used as a reference genome), as identified by BLASTp searches of the proteins encoded by each SAG. The amino acid identities of proteins encoded by the SAGs relative to proteins encoded in the NSHQ14C MAG were then averaged to calculate an overall average amino acid identity for each SAG.

Orthologous protein groups among the SAGs were first defined as those proteins encoded in SAGs that were most closely related to a single protein from the reference genome (NSHQ14C Type II MAG), as determined by BLASTp. Among the most commonly identified SAG orthologs that exhibited differences in pairwise amino acid identity were transposases. Transposase orthologs were further classified using ACLAME v.0.4 [61] and were then subjected to alignment with Clustal Omega and maximum-likelihood phylogenetic reconstruction with PhyML v.3.0 via the Bayesian Information Criterion as implemented in the Smart Model Selection in PhyML [62, 63]. The synteny of genes flanking transposases was assessed by aligning contigs encoding transposases using progressiveMauve [64], and alignments were visualized using Gene Graphics [65]. To evaluate the possibility that divergent orthologous proteins contributed to apparent diversification of SAGs, Mantel tests describing correlations between protein ortholog amino acid dissimilarities and whole genome ANI dissimilarities were performed for the ten most abundant orthologous proteins identified in SAGs that differ from those in the NSHQ14 Type II MAG. Mantel tests were performed using the mantel function of the vegan: community ecology package in R specifying 100 permutations using the Pearson correlation method [66].

### Methanogenesis rate potentials

Potential rates of biological transformation of HCO_3_^−^ and HCOO^−^ to CH_4_ were measured via microcosm-based activity assays containing ^14^C-radiolabeled substates as previously described [18]. Briefly, well water samples stored at 4 °C were homogenized and 10 mL aliquots were transferred from storage vials to 26 mL N_2_-purged, autoclave-sterilized, butyl-stoppered experimental vials (four biological replicates and four abiotic controls per condition) using a sterile, N_2_-purged needle and syringe. Abiotic controls were sterilized by autoclaving twice with incubation at room temperature overnight between autoclave cycles to allow for germination of spores. 1 mM (final concentration) of HCOO^−^ or HCO_3_^−^ that included 5 µCi ^14^C-HCOO^−^ or ^14^C-HCO_3_^−^ (American Radiolabeled Chemicals, St. Louis, MO), respectively, was then added to each vial. H_2_ gas was added in excess of atmospheric pressure to each vial to account for 20% of headspace volume. Following incubation for 1, 2, 4, 6, and 8 weeks at in situ temperature (34 °C), 3 mL filtered (0.2 μm) N_2_ was added to each vial using an N_2_-purged sterile needle and syringe to maintain positive headspace pressure, and 3 mL headspace gas was removed using a needle and syringe equipped with a stopcock. The 3 mL headspace gas from each microcosm vial was then injected into a specially fabricated scintillation vial containing a butyl rubber septum and CH_4_ was trapped with 10 mL Cytoscint ES scintillation cocktail, as described previously [18]. The radioactivity, measured in counts per minute, associated with ^14^C-CH_4_ from each of the samples was measured on a PerkinElmer Tri Carb 2900TR Liquid Scintillation Analyzer (PerkinElmer, Waltham, MA), converted to disintegrations per minute using a quench curve, normalized to account for total substrate added (radiolabeled and unlabeled) and total headspace gas volume, and used to calculate the rates of substrate transformation. ^14^C-CH_4_ values in abiotic controls were subtracted from ^14^C-CH_4_ values in biological replicates to arrive at the amount of ^14^C-CH_4_ produced that could be attributed to biology. Statistical significance of differences between biological assays and abiotic controls were assessed via Student’s *t-*test assuming unequal variance for each condition.

## Results and discussion

**Fig. 1:**
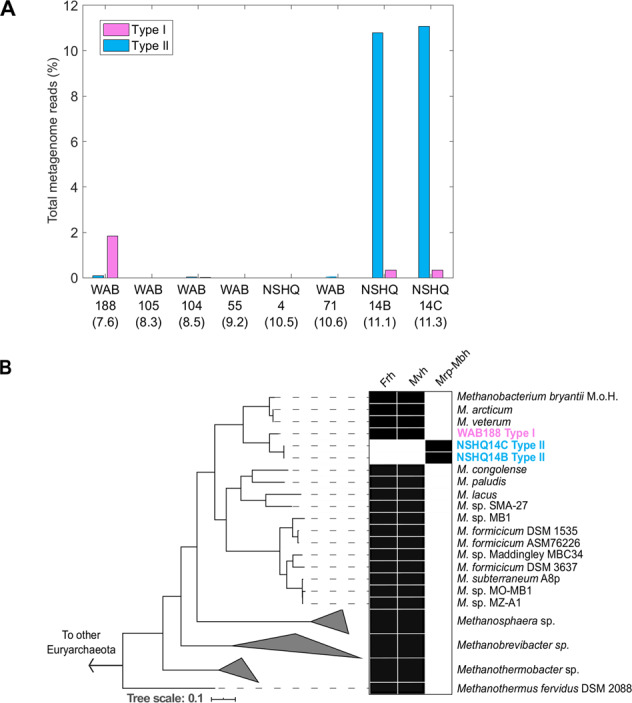
The estimated relative abundances of *Methanobacterium* MAGs in communities from subsurface fracture waters collected from wells intersecting the Samail Ophiolite in 2017 (A) and phylogenomic reconstruction of Oman methanogen metagenome assembled genomes (MAGs) in relation to representative *Methanobacterium* genomes (B). In **A**, wells are listed in order of ascending pH from left to right with pH values indicated below the name of each well in parentheses. Contigs with ≥98% nucleotide identity to the *Methanobacterium* Type I MAG from WAB188 were classified as Type I and contigs with ≥98% nucleotide identity to the *Methanobacterium* Type II MAG from NSHQ14C were classified as Type II. Estimated relative abundances are shown as the number of reads affiliated with *Methanobacterium* Type I or II MAGs as a percentage of the total reads in each metagenome. In **B**, pink text depicts the Type I MAG and cyan text depicts Type II MAGs. All bootstrap values of displayed nodes are >980 out of 1000 bootstrap replicates. Clade-level triangles indicate the phylogenetic diversity within each group via side lengths that are proportional to the distances between the clade’s most closely related and furthest related taxa. Branch length is relative to the scale provided at the bottom of the figure indicating the expected number of substitutions per site. Filled boxes to the right of terminals indicate the presence of genes in reconstructed MAGs or genomes, whereas empty boxes indicate absence of genes in reconstructed MAGs or genomes. *Frh* coenzyme F_420_-reducing (Group 3a) [NiFe]-hydrogenase, *Mvh* methyl viologen (Group 3c) [NiFe]-hydrogenase, *Mrp-Mbh* multiple resistance and pH adaptation module—membrane-bound (Group 4) [NiFe]-hydrogenase protein complex.

Reconstructed genomes from metagenomic sequences were used to develop metabolic models for methanogen populations inhabiting waters that span a range of geochemical conditions due to differences in serpentinization reaction progress. Two phylogenetically distinct lineages affiliated with *Methanobacterium* were the sole methanogens recovered from subsurface well waters from the Samail Ophiolite (Fig. 1). One MAG (Type I) represented an abundant microbial lineage in a well with circumneutral water (WAB188: pH 7.6), whereas another MAG (Type II) represented a lineage that was abundant in a well with hyperalkaline water, including samples collected at both 50 m (NSHQ14B: pH 11.1) and 85 m depths (NSHQ14C: pH 11.3) (Fig. 1A). MAGs affiliated with the Type II lineage from NSHQ14B and from NSHQ14C exhibited >99.99% identity to each other on an average nucleotide level (data not shown). Due to the extremely high similarity between these two MAGs, one MAG (*Methanobacterium* Type II from NSHQ14C) was selected as the reference genome for the Type II lineage. Contigs affiliated with both Type I and Type II lineages were also detected at low abundances in most of the other wells, which is consistent with the previous finding that 16S rRNA genes affiliated with *Methanobacterium* were widely distributed at varying abundances among subsurface well waters collected from the Samail Ophiolite (e.g., previously detected in 16 of 20 samples in relative abundances of <3% to 28.0% of total 16S rRNA gene transcripts) [19]. Accordingly, sequences closely affiliated with *Methanobacterium* have been detected in a range of other serpentinites and often in high abundance [13, 14, 23], suggesting that this genus is a ubiquitous member of globally distributed serpentinite communities.

All *Methanobacterium* MAGs exhibited high levels of estimated completeness and low contamination (Supplementary Table S1), and were classified as high-quality draft genomes [67]. The Type I *Methanobacterium* MAG was larger (1.904 Mbp) than the NSHQ14C *Methanobacterium* Type II MAG (1.511 Mbp) and encoded more proteins (2052 versus 1806), despite both MAGs exhibiting the same estimated levels of completeness (98.4%) (Supplementary Table S1). This finding is consistent with previous results that revealed an inverse relationship between the average estimated genome size and the pH of subsurface well waters in the Samail Ophiolite [18]. This observation is also consistent with data from the serpentinization impacted environment termed The Cedars (California, USA), where members of a microbial community from a hyperalkaline spring (pH ~12) harbored the smallest genomes reported for their respective taxa [68]. Microorganisms residing in highly serpentinized waters presumably exhibit streamlined genomes to minimize energetic costs and nutrient demands associated with their replication and repair under extreme conditions, as has been suggested previously [18, 69].

Phylogenomic reconstruction of Oman methanogen MAGs in relation to other *Methanobacterium* genomes suggested that the taxa corresponding to Type I and II MAGs diverged relatively recently within the *Methanobacterium* lineage (Fig. 1B). The Type I and II MAGs were most closely related to each other, and unexpectedly, the Type II MAG appeared to correspond to a taxon that was descended from the Type I taxon, as described below. The Type I MAG encoded homologs of two key [NiFe]-hydrogenases, Group 3c bifurcating [NiFe]-hydrogenase (Mvh) and Group 3a F_420_-reducing [NiFe]-hydrogenase (Frh) (discussed below) (Figs. 1B; S1), which are common to other Methanobacteria. However, homologs of these genes were not detected in any of the Type II genomes, including both Type II MAGs and all single amplified genomes (SAGs, described below). Furthermore, the large subunits of Group 3 [NiFe]-hydrogenases encoded by the Type I MAG were used to query the entire assemblies of both NSHQ14 metagenomes with no positive matches identified. Together, these data indicated gene loss in the Type II lineage. In contrast, among available Methanobacteria genomes, only the Type II lineage encoded a homolog of the ion-translocating [NiFe]-hydrogenase complex, Mrp-Mbh, indicating gene acquisition (Figs. 1B;  S2). The large subunit of the Mrp-Mbh [NiFe]-hydrogenase encoded by the Type II MAGs was not detected in the WAB188 metagenomic assembly. Phylogenetic analysis of the large subunit of the Mrp-Mbh [NiFe]-hydrogenase encoded by the Type II MAGs revealed it to be nested among bacterial Group 4d [NiFe]-hydrogenase homologs, suggesting acquisition via horizontal gene transfer from an ancestor of the Firmicutes or Thermotogae (Supplementary Table S2**;** Supplementary Fig. S2). Members of the Firmicutes are commonly identified in subsurface waters of the Samail Ophiolite [19] and other serpentinizing systems [14, 23], leading to the possibility that the genes encoding Mrp-Mbh could have been acquired in a similar environment type. As such, the divergence between Type I and Type II MAGs appears to have occurred relatively recently, a conclusion that is supported by short estimated branch lengths separating these lineages (Fig. 1B).

**Fig. 2:**
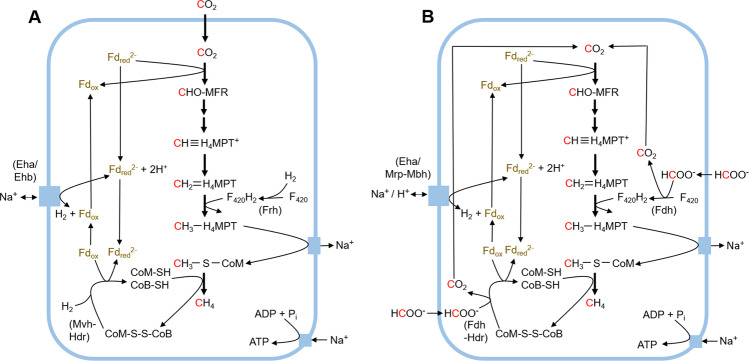
Proposed hydrogenotrophic and formatotrophic methanogenesis pathways in *Methanobacterium* Type I (A) and Type II (B) populations, respectively, from the Samail Ophiolite, Oman. Enzymes that differed between the two MAGs are denoted in the figures. *Eha* Group 4d energy-converting [NiFe]-hydrogenase A, *Ehb* Group 4d energy-converting [NiFe]-hydrogenase B, *Fd* ferredoxin, *Fdh* formate dehydrogenase, *Frh* coenzyme F_420_-reducing (Group 3a) [NiFe]-hydrogenase, *Hdr* heterodisulfide reductase, *MFR* methanofuran, *MPT* methanopterin, *Mrp-Mbh* multiple resistance and pH adaptation module—membrane bound (Group 4) [NiFe]-hydrogenase protein complex, *Mvh* methyl viologen-reducing (Group 3c) [NiFe]-hydrogenase. Figure adapted from Boyd et al. [1].

Metabolic reconstructions of *Methanobacterium* Type I and II MAGs indicated that both organisms encoded methanogenesis pathways, albeit with several key differences as mentioned briefly above (Fig. 2). The Type I MAG encoded all requisite genes for hydrogenotrophic methanogenesis in Methanobacteria [70] (Fig. 2A), including Group 4 energy-converting [NiFe]-hydrogenase (Eha/Ehb), Frh, and the Mvh-heterodisulfide reductase complex (Mvh-Hdr) (Supplementary Figs. S1;  S2). Homologs of H_2_-dependent methenyltetrahydromethanopterin dehydrogenase (Hmd) and F_420_-dependent methylenetetrahydromethanopterin dehydrogenase (Mtd), which can function independently or in tandem to catalyze a critical step in hydrogenotrophic methanogenesis [71, 72], were initially not detected in either the Type I or Type II MAG. However, contigs containing homologs of Mtd with 89% identities to a previously described *Methanobacterium* sp. per BLASTp were later identified in unbinned contigs of the WAB188 metagenome and both NSHQ14 metagenomes and were assigned to the Type I and Type II MAGs, respectively (Supplementary Table S3).

The Type II MAG encoded Eha (Supplementary Figs. S2; S3) but surprisingly did not encode Frh (Fig. 1B; Supplementary Table S4), the latter of which couples the oxidation of H_2_ to the reduction of coenzyme F_420_ [56, 73]. The Type II MAG also did not encode Mvh (Fig. 1B; Supplementary Table S4), which couples with Hdr to bifurcate electrons from H_2_ to simultaneously reduce low potential ferredoxin (Fd) and the heterodisulfide bond between coenzyme M and coenzyme B (CoM-S-S-CoB) [56]. Mvh-Hdr and Frh are [NiFe]-hydrogenases characteristically encoded in the genomes of all other members of the Methanobacteria (Fig. 1B; Supplementary Table S4) and are common features associated with all hydrogenotrophic (Class I) methanogens [70]. Importantly, genetic experiments indicated that neither Mvh nor Frh homologs are required during methanogenic growth with formate [74, 75]. For example, in *Methanococcus maripaludis* Vhu (alternative name for Mvh) and Frh deletion strains, these functions are thought to be replaced by a formate dehydrogenase (Fdh)–Hdr complex and a F_420_-reducing Fdh, respectively [74–78]. FdhAB and VhuD proteins have been suggested to form a complex with HdrABC that allows for bifurcation of electrons from formate and their coupled reduction of heterodisulfide and Fd [76, 79]. In support of this, the alpha subunit of Fdh from *M. maripaludis* co-purified with the beta subunit of Fdh, the delta subunit of Vhu, and all subunits (i.e., ABC) of Hdr [76, 79]. In addition, the tungsten-containing formylmethanofuran dehydrogenase, which catalyzes the reduction of CO_2_ and covalent attachment of methanofuran in the first step of methanogenesis, was found to co-purify with FdhAB, HdrABC, and VhuD in *M. maripaludis* when grown under formatotrophic, but not hydrogenotrophic, conditions [76]. This may help to explain how *M. maripaludis*, and potentially other hydrogenotrophic methanogens, prevent the loss of cytoplasmic CO_2_ for use in methanogenesis and biomass generation during formate-dependent growth.

**Fig. 3:**
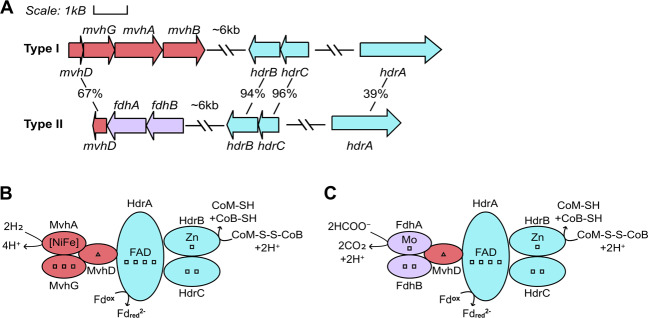
Genes inferred to be co-localized with those coding for methyl viologen-reducing (Group 3c) [NiFe]-hydrogenase (Mvh) and formate dehydrogenase (Fdh) in Type I and II MAGs (A) and the putative protein complexes they form to bifurcate electrons from H_2_ (B) or formate (C), respectively, to simultaneously reduce ferredoxin and heterodisulfide. Percentages below each gene indicate amino acid identities between homologs encoded by Type I and Type II MAGs. In panels B and C, squares represent [4Fe-4S] clusters and triangles represent [2Fe-2S] clusters. *CoB* coenzyme B, *CoM* coenzyme M, *FAD* flavin adenine dinucleotide, *Fd* ferredoxin, *Fdh* formate dehydrogenase, *Hdr* heterodisulfide reductase, *Mvh* methyl viologen-reducing (Group 3c) hydrogenase. Figure modified from Thauer et al. [71] and Costa et al. [77].

The biochemical and phenotypic observations of Frh and Vhu deletion strains of *M. maripaludis* provide a framework to rationalize how the Type II cells from the Samail Ophiolite, which lack Frh and Mvh, might catalyze methanogenesis. The Type II MAG encoded Fdh (*fdhAB*) and this was inferred to be co-localized in the genome with *mvhD* and *hdrBC* (Fig. 3). While the structure of F_420_-reducing Fdh has not yet been resolved [77], Fdh purified from *Methanobacterium formicicum* reduced coenzyme F_420_ [80, 81], and the beta subunit (FdhB) was predicted to have an F_420_-binding domain similar to that of the Frh beta subunit (FrhB) [82]. The residues involved in F_420_ coordination by FrhB in *Methanothermobacter marburgensis* (positions 163, 165–167, and 208–211) [82] were conserved in FdhB proteins from both *M. formicicum* and the Type II *Methanobacterium* MAG with two exceptions (Supplementary Fig. S4). The two positions that were not conserved in *M. formicicum* and the Type II MAG, however, harbor substitutions to amino acids of the same polarity and charge (S to N and V to W) and were consistent between both FdhB proteins. The mechanism of formate transport into the cell remains somewhat unclear as *Methanobacterium* Type I and Type II MAGs both lacked homologs of the canonical formate transporters FdhC, YfdC, and FocA. Each *Methanobacterium*-affiliated SAG and the entire metagenomic assemblies from WAB188, NSHQ14B, and NSHQ14C were queried for these possible formate transporters with no positive matches identified. However, the Type II MAG encoded a homolog (38.8% identity, 95% query coverage) of the formate:oxalate antiporter, OxlT (Supplementary Table S2), which was lacking from the Type I MAG and may function in transporting formate into the Type II cells. These data collectively suggest that the Type II *Methanobacterium* may drive methanogenesis via a pathway that is unique among characterized Methanobacteria, wherein formate is required as electron donor and with H_2_ serving as a source of additional reductant (Fig. 2B).

Consistent with previous work suggesting an essential anaplerotic role for Eha during formatotrophic methanogenesis [74], both the Type I and II MAGs encoded homologs of this enzyme complex (Supplementary Figs. S2, S3). The Type II MAGs, but not Type I MAG or other members of the Methanobacteria (Fig. 1B), also encoded a [NiFe]-hydrogenase that is homologous to the membrane-bound Mrp-Mbh complex in *Pyrococcus furiosus* (Supplementary Fig. S5) [83]. Mrp-Mbh in *P. furiosis* comprises 14 subunits, including a Na^+^/H^+^ antiporter domain (Mrp) and a [NiFe]-hydrogenase domain (Mbh) [83]. In *P. furiosus*, the Mrp–Mbh complex catalyzes the reversible oxidation of Fd coupled with H_2_ production, with excess potential used to pump Na^+^ or H^+^ outside of the cell. In this case, oxidation of Fd would generate an electrochemical gradient that could be used to drive ATP synthesis. Alternatively, Fd reduction could be coupled to H_2_ oxidation, with the shortage of potential compensated for by releasing Na^+^ or H^+^ into the cell. Consequently, the Type II *Methanobacterium* cells could use this complex to: (1) generate reduced Fd and (2) neutralize cytoplasmic pH (if the coupling ion is H^+^). Indeed, Mrp was first characterized in the alkaliphile *Bacillus halodurans*, where it was found to be critical for pH homeostasis under alkaline conditions [84]. The reversibility of the enzyme system may allow for these functions to shift with changing cellular demands.

Why would *Methanobacterium* Type II have evolved to drive methanogenesis with formate rather than H_2_ as primary reductant in an environment such as NSHQ14, where H_2_ concentrations (21–2900 μM) were over 1–3 orders of magnitude higher than formate (1.7 μM) (Table 1) [18, 19, 25]? The standard state reduction potentials of hydrogen and formate are similar (−414 and −420, respectively) [85], although the nonstandard state potential of H_2_ is likely to be lower than that of formate in environments where the concentration of H_2_ exceeds formate. It is also possible that the differential mobility of formate and H_2_ in aqueous solutions could favor formate utilization [86]. Alternatively, we propose adaptation to extreme CO_2_ limitation in highly serpentinized waters as a likely explanation. Although H_2_ was replete in the environment inhabited by Type II *Methanobacterium* cells, use of H_2_ as sole electron donor for methanogenesis necessitates coupling with CO_2_ as both electron acceptor and source of carbon [70]. However, dissolved CO_2_ is extremely limited in highly serpentinized waters due to prior reactions that precipitated DIC as mineral carbonates. Furthermore, DIC speciates primarily as bicarbonate or carbonate ions under high pH conditions such as those present in NSHQ14 [8]. Although methanogens may convert bicarbonate to CO_2_ for use in methanogenesis or biomass generation, this reaction (HCO_3_^−^ + H^+^ → CO_2_ + H_2_O) consumes protons, which are limiting at high pH. Formatotrophic methanogenesis may help circumvent carbon acquisition problems because the oxidation of formate yields intracellular CO_2_ which could be subsequently reduced to CH_4_ (Fig. 2B) or biomass via the Wood-Ljungdahl pathway (Table S2). In addition, a putative acetogen lacking all known hydrogenases and apparently using CO to drive acetogenesis was detected in the CO_2_-limited, serpentinization-impacted groundwater of The Cedars, California [87]. This suggests that replacement of hydrogen-based reductant by single-carbon reduced substrates (i.e., formate or CO) may be a common strategy to circumvent CO_2_ limitation among organisms encoding the Wood–Ljungdahl pathway of carbon fixation in serpentinizing environments.

The source of formate in waters of the Samail Ophiolite has not yet been resolved, however, consumption of CO_2_ and H_2_, coinciding with generation of nearly 100 μM formate, has been observed during low-temperature abiotic reactions between water and rocks from the Samail Ophiolite [38]. A positive correlation (Pearson *R* = 0.72, *p* < 0.05) existed between formate concentrations and pH in subsurface waters from the Samail Ophiolite sampled in 2015 [19] (formate was not measured in 2017), which is potentially consistent with the hypothesis that formate can be generated abiotically as a result of equilibration of CO_2_ with H_2_ produced during serpentinization reactions [8, 9, 88, 89]. In addition, formate formed via past reactions could potentially be stored in the rock (e.g., in fluid inclusions) [90]. Regardless of the source of formate in the system, it is possible that methanogens and other formatotrophic populations actively maintain formate at a low (~1 µM) steady state concentration (Table 1).

**Fig. 4:**
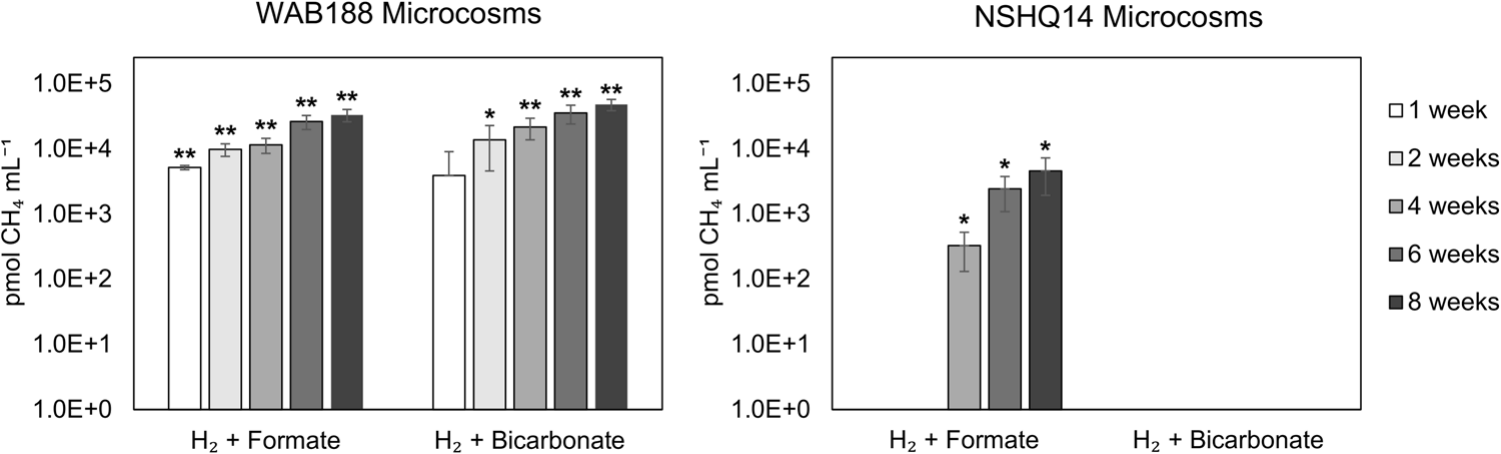
Potential rates of biological methanogenesis from formate and bicarbonate by planktonic microbial communities in well water samples collected from the Samail Ophiolite in 2020. Potential rates of biological substrate transformation were determined via microcosm assays using well waters collected from the Samail Ophiolite in 2020. Results are plotted on a logarithmic scale. The average rates of methane generation observed in four replicate abiological controls were subtracted from values in four replicate biological assays (Avg) and their combined standard deviations (SD) are presented at five timepoints over an 8-week incubation. *P*-values were determined between biological assays and abiological controls at each timepoint via Student’s *t*-test assuming unequal variance for each condition (*p < 0.05, **p < 0.01).

To begin to test these predictions, well waters were sampled again from WAB188 and NSHQ14 in February and March, 2020, for use in quantifying rates of ^14^C-CH_4_ generation from ^14^C-radiolabeled substrates (^14^C-HCOO^−^ and ^14^C-HCO_3_^−^). Temperature and pH values were similar between waters sampled from each well in 2017 and 2020 (Table 1). Microcosms containing waters from WAB188 that were amended with either H_2_ + bicarbonate (including 5μCi ^14^C-HCO_3_^−^) or with H_2_ + formate (including 5 μCi ^14^C-HCOO^−^) generated significantly more CH_4_ than killed controls, and CH_4_ generation attributable to biological processes increased over time (Fig. 4, Supplementary Table S5). No significant differences were observed between the amount of biologically-generated CH_4_ in WAB188 microcosms amended with H_2_ + bicarbonate versus H_2_ + formate at each time point. This is potentially consistent with the hypothesis that *Methanobacterium* Type I, which was detected in high abundance in well WAB188 among 2017 metagenomes, functions as a canonical Class I methanogen (similar to other members of the Methanobacteria) which can commonly use H_2_ + CO_2_ or formate as methanogenic substates [70].

In contrast, significantly higher quantities of CH_4_ were generated in NSHQ14 microcosms amended with H_2_ + formate (including 5μCi ^14^C-HCOO^−^) than in killed controls, and biologically-generated CH_4_ increased over time (Fig. 4, Supplementary Table S5). However, no CH_4_ generation attributable to biology was observed in NSHQ14 microcosms amended with H_2_ + bicarbonate (including 5μCi ^14^C-HCO_3_^−^). This potentially supports the genomic prediction that *Methanobacterium* Type II, which was detected in high abundance in NSHQ14 among 2017 metagenomes, can couple H_2_ with formate but not DIC to drive methanogenesis via a pathway that is unique among characterized Methanobacteria. Further, the rates of methanogenesis were lower in NSHQ14 microcosms as compared with WAB188 microcosms, which is potentially consistent with the previous finding that rates of utilization of select single-carbon compounds were generally lower in hyperalkaline waters than alkaline waters, possibly due to extreme conditions imposed by high pH waters [18].

The short branch length separating the Type I and Type II MAGs and differences in their methanogenesis pathways due to gene loss/acquisition suggests that the Type II cells were derived from the Type I lineage. This prompted the generation of single amplified genomes (SAGs) to further evaluate whether this phenomenon was prevalent among individual cells and to uncover evidence for continued strain-level diversification in this lineage. A total of 71 SAGs were assembled from NSHQ14C, 69 of which were affiliated with *Methanobacterium* (Supplementary Table S6). One of the other SAGs was affiliated with the methanotrophic bacterial genus, *Methyloccocus*, and the other was too incomplete to classify taxonomically. The low genome recovery from SAGs (71 out of 317 single cells) is possibly attributable to the low efficiency of lysing cells that are adapted to hyperalkaline waters using potassium hydroxide.

**Fig. 5:**
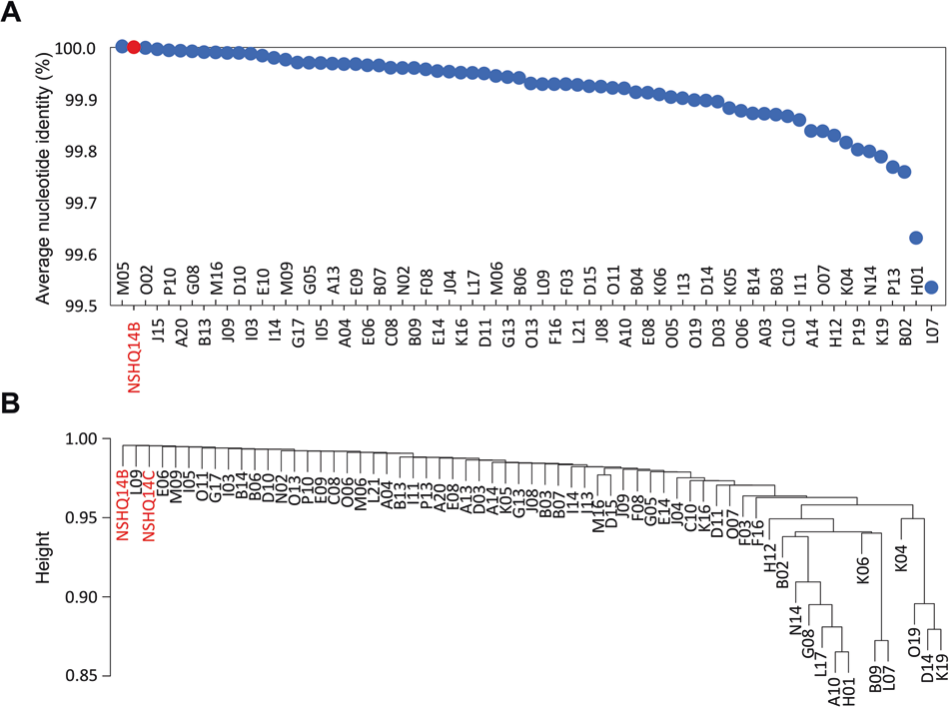
Average nucleotide identities (ANIs) between *Methanobacterium* single amplified genomes (SAGs) and Type II metagenome assembled genomes (MAGs). In **A**, ANIs relative to the NSHQ14C MAG are presented with blue circles representing individual SAGs and the red circle representing the NSHQ14B MAG. In **B**, pairwise ANIs among the SAGs and the NSHQ14C MAG were used to generate a dendrogram based on hierarchical clustering, with branch lengths (“Height”) representing Euclidean distances between ANIs calculated among the genomes. Red text represents the Type II MAGs and letters followed by two-digit numbers represent individual SAGs.

Among the 69 *Methanobacterium* affiliated SAGs, the pairwise average nucleotide identities (ANIs) indicated an average difference of only ~1 bp per kbp between them and the NSHQ14C Type II MAG (average ANI of 99.90%) (Fig. 5A) and average amino acid identities between the SAGs and the NSHQ14C Type II MAG ranged from 95 to 100% (Supplementary Fig. S6). For comparison, the Type I MAG shared 91.11% and 90.59% ANI with the Type II MAGs from NSHQ14B and NSHQ14C, respectively. Proteins involved in cellular metabolism (e.g., HdrABC, FdhB, MvhD, and the large subunits of both Eha and Mrp–Mbh [NiFe]-hydrogenases) were all found to exhibit identical nucleotide sequences among the Type II SAGs and MAGs. Similarly, FdhA exhibited identical nucleotide sequences among all Type II SAGs and MAGs, except for a single SAG (B06) that was found to exhibit a single non-synonymous nucleotide polymorphism resulting in a change from an aspartate to a glutamate. Both aspartate and glutamate have negatively charged side chains, indicating that this change likely conserved protein function, which is consistent with the presumed critical importance of FdhA in the metabolism of Type II cells (Fig. 3B). Consistent with the Type II MAGs, homologs of Mvh and Frh [NiFe]-hydrogenases were not found in any of the *Methanobacterium* affiliated SAGs. Together, these observations indicate that the core metabolism inferred for the Type II cells is likely a prevalent feature associated with this population.

In contrast, a number of orthologous proteins were identified in SAGs that differed markedly from those encoded by the NSHQ14C Type II MAG. Notably, among the ten orthologs that had the highest representation in SAGs and that differed from the NSHQ14C MAG, five were transposases (Table 2). This finding is in line with the previous detection of abundant transposases in a biofilm metagenome recovered from the Lost City serpentinizing system [91]. Mantel tests of matrices describing the ANI among SAGs (Supplementary Table S7) and the amino acid dissimilarity of the ten orthologs (Table 2) were used to identify orthologs that likely contributed to apparent diversification in the SAGs (Fig. 5). Among the ten orthologs considered, the dissimilarities in eight proteins were not significantly correlated with ANI. However, the dissimilarity of a protein belonging to the Peptidase C39 family and an ISNCY (“insertion sequence not classified yet”) transposase were significantly and positively correlated with the dissimilarity in ANI, suggesting that they may contribute to the apparent recent diversification of these cells.

**Table 2:** The ten most abundant orthologous proteins identified in single amplified genomes (SAGs) that differ from those in the NSHQ14C Type II metagenome assembled genome (MAG).

Variant protein ortholog annotation (SAGs vs. NSHQ14C MAG)	# of SAGs encoding orthologs with <100% identity to the NSHQ14C MAG	Amino acid identities (SAGs vs. NSHQ14C MAG)	Closest taxonomic affiliation of the most divergent ortholog	Mantel correlation (R) and significance (*p*) among protein ortholog amino acid dissimilarities and whole genome ANI dissimilarities
Transposase	21	96.8–98.4%	*Methanosarcina mazei*	*R* = −0.01, *p* = 0.44
GDP-mannose 4,6 dehydratase	17	73.9–100%	*Methanobacterium congolense*	*R* = 0.07, *p* = 0.07
DDE-type integrase/ transposase/recombinase	17	97.2–100%	*Methanobacterium formicicum*	*R* = 0.09, *p* = 0.21
Peptidase C39	16	76.5–100%	*Methanothermus fervidus*	*R* = 0.19, *p* = 0.02
Transposase (IS5 family)	16	96.6–100%	*Methanoculleus taiwanensis*	*R* = 0.04, *p* = 0.23
ISNCY family transposase	15	91.6–100%	*Methanobacterium subterraneum*	*R* = 0.37, *p* = 0.01
Histidine kinase	13	99.7–100%	*Methanobacterium formicicum*	*R* = 0.22, *p* = 0.07
Transposase (IS630 family)	12	97.4–100%	*Methanobacterium subterraneum*	*R* = −0.08 *p* = 0.84
MFS transporter	12	99.8–100%	*Methanobacterium paludis*	*R* = 0.01 *p* = 0.43
Exosome complex protein Rrp4	7	99.6–100%	*Methanobacterium congolense*	*R* = 0.01 *p* = 0.50

**Fig. 6:**
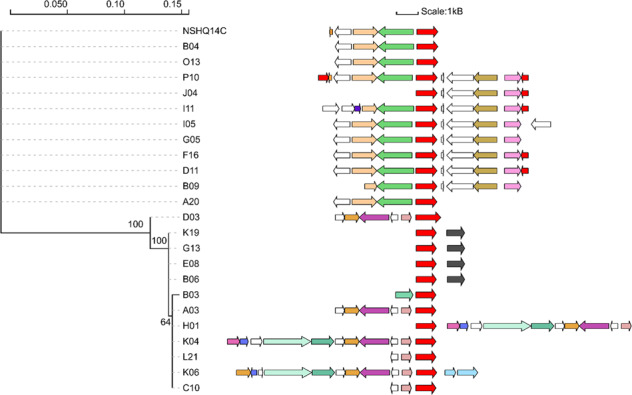
ISNCY transposase protein phylogeny and genes co-localized with this transposase in the NSHQ14C metagenome assembled genome (MAG) and single cell genomes (SAGs). Branch length is relative to the scale provided at the top of the figure indicating average substitutions per site. Bootstrap values are displayed at each node (out of 100 replicates). Contigs that encoded this transposase in each SAG or MAG are depicted to the right of the terminals. Red arrows represent genes encoding transposase orthologs, white arrows represent genes encoding hypothetical proteins, black arrows represent genes encoding CRISPR-associated (Cas) proteins, and the remaining colored arrows represent genes encoding other functional proteins. Gene lengths are relative to the scale provided at the top of the figure.

The genes flanking the ten divergent orthologs were examined to identify evidence of recent transposition and/or horizontal gene transfer events in the form of different genomic locations in the SAGs. The unclassified transposase with the greatest number of SAG homolog variants that, based on the results of a Mantel test (Table 2), is not suggested to have significantly contributed to the recent vertical diversification of SAGs, was flanked by a randomized assortment of genes (data not shown). This is consistent with its recent and likely random integration into the genomes of Type II cells. In contrast, genes flanking the ISNCY family transposase that is suggested to have significantly contributed to recent diversification of these cells were largely syntenic among closely related orthologs but not among those encoding more divergent orthologs (Fig. 6). In addition, variability of gene order and gene content among the SAGs and MAGs suggested rearrangement of genes flanking this transposase. These observations suggest that multiple transposition events likely led to this variable genomic arrangement, thereby revealing a mechanism that could have contributed to the recent diversification of Type II cells.

Intriguingly, SAGs K19, G13, E08, and B06 all encoded closely related ISNCY transposases upstream of a CRISPR-associated Cas4 protein (Fig. 6). This Cas4 protein was not encoded by the Type II NSHQ14B or NSHQ14C MAGs, with the most similar protein in the MAGs only exhibiting <30% identity. Moreover, this protein was only encoded by these four SAGs and one other (D03) where it was located on a contig that encoded multiple putative transposases (data not shown). Surprisingly, however, the contigs encoding Cas4 and the ISNCY transposase did not code for other Cas proteins or other genetic elements (i.e., CRISPRs and spacer sequences) that act in tandem with Cas4 [92] to confer adaptive immunity against invading viruses and plasmids in Bacteria and Archaea [93]. However, Cas4 is not always directly colocalized with other elements of CRISPR arrays [94] and, importantly, the ends of contigs represent portions of the genome where reads do not sufficiently overlap to generate contiguous sequences. Therefore, it is possible that transposases (especially ISNCY type) catalyzed the integration of functional CRISPR-Cas systems that may confer a subset of the population with adaptive immunity from foreign genetic material.

The findings herein are potentially in line with previous studies of organisms that are closely related to one another, wherein microbial genomes have been conceptualized as containing two distinct components, core and variable, collectively termed the pan-genome [95, 96]. The core genome is composed of genes that are common to all strains, whereas the variable genome consists of genes that differ between strains, for example resulting from gain via horizontal gene transfer or loss. For example, seven *Sulfolobus islandicus* genomes recovered from globally distributed locations that diverged recently in evolutionary time (~910,000 years ago) shared a core genome including housekeeping genes, but also encoded variable genomic regions containing small inversions and rearrangements, many of which were also associated with CRISPR-Cas genes [95]. Like the *Methanobacterium* populations from 85 m depth in NSHQ14, other subsurface communities include population level genomic diversity that was proposed to have been generated via the activity of mobile elements [97–99].

Similar to the ISNCY transposase, genes flanking a protein belonging to the C39 peptidase family that is suggested to have contributed to recent diversification of Type II *Methanobacterium* cells (Supplementary Table 2) were largely syntenic among closely related proteins but not among those encoding more divergent orthologs, and rearrangement of genes co-localized with this peptidase were also observed (Supplementary Fig. S7). These rearrangements may have been catalyzed by a transposase that was detected downstream of the peptidase in at least one of these SAGs (I11). The diversification of peptidase C39 and its potential integration into cellular genomes by transposase activity may have implications for cellular fitness as these proteins function in bacteriocin (antimicrobial peptide) processing [100]. Thus, it is possible that Type II *Methanobacterium* cells employ bacteriocidin-type antimicrobials to increase their competitive advantage for limiting nutrients (e.g., formate) over other co-inhabiting species, potentially helping to explain their prevalence in hyperalkaline waters.

## Conclusions

Reconstruction of two *Methanobacterium* genomes from metagenomic sequences obtained from subsurface waters exhibiting contrasting geochemical characteristics of the Samail Ophiolite, Sultanate of Oman, provided an opportunity to conduct comparative genomic and evolutionary investigations into adaptations that enable putative hydrogenotrophic methanogens to overcome DIC limitation associated with hyperalkaline conditions. Metabolic reconstruction of a Type I *Methanobacterium* MAG that was abundant in circumneutral, DIC replete waters revealed canonical hydrogenotrophic methanogenesis pathways. However, Type II *Methanobacterium* MAGs from extensively serpentinized, DIC limited waters lacked homologs of key [NiFe]-hydrogenases, Mvh and Frh, that supply reductant from H_2_ oxidation for methanogenesis. Metabolic reconstructions indicated that these functionalities were replaced by formate dehydrogenases that supply reductant from formate oxidation and that yield intracellular CO_2_ to allow for methanogenesis to proceed under otherwise DIC limited conditions. The genomic prediction that *Methanobacterium* Type II can couple H_2_ with formate but not DIC to drive methanogenesis via a pathway that is unique among characterized Methanobacteria was supported by microcosm-based radiotracer experiments revealing significant biological methane production from H_2_ + formate but not H_2_ + bicarbonate. The combination of phylogenetic and gene distribution data relative to other Methanobacteria indicated that the Type II lineage was derived from the Type I lineage, suggesting that the replacement of H_2_-based reductant by formate-based reductant is a derived trait. In turn, this suggested that the directionality of the diversification of *Methanobacterium* inhabiting the Samail Ophiolite was from circumneutral into hyperalkaline environments, the latter of which is likely to be limiting in DIC, but where formate could be supplied by serpentinization reactions. Thus, diversification may have taken place in a system where serpentinization was generating large amounts of formate which could act as a selective pressure to evolve the suite of physiological traits allowing for its use in key methanogenesis reactions.

The recent diversification of *Methanobacterium* into hyperalkaline environments appeared to have been facilitated, in part, by lateral gene transfer of a [NiFe]-hydrogenase complex from a bacterium, perhaps affiliated with an ancestor of Firmicutes. Single cell genomes of Type II *Methanobacterium* revealed evidence of gene rearrangement, possibly through transposition. Collectively, these results point to the importance of gene loss, gain, and transposition in the adaptation of methanogens to hyperalkaline conditions and provide strong, albeit indirect, evidence that cells are active and adapted to the polyextremophilic subsurface environment of the serpentinizing Samail Ophiolite.

## Supplementary information

Supplemental Material

Supplemental Figure 2

Supplemental Table 2

Supplemental Table 4

Supplemental Table 6

Supplemental Table 7
