# Supplementary material for: Diversification of methanogens into hyperalkaline serpentinizing environments through adaptations to minimize oxidant limitation
Source: ISME J. 2020 Nov 30;15(4):1121–35. doi: 10.1038/s41396-020-00838-1 (PMC8115248; doi:10.1038/s41396-020-00838-1)
Supplement: Supplementary file 1 — Supplemental Material [file 41396_2020_838_MOESM1_ESM.pdf]

SUPPLEMENTARY MATERIALS

Supplementary Table 1. Assembly statistics for *Methanobacterium* Type I and II metagenome assembled genomes (MAGs).

	Methanobacterium Type I	*Methanobacterium* Type II
Bins	WAB188.10	NSHQ14B.3 / NSHQ14C.4
Size (Mbp)	1.904	1.496 / 1.511
Completeness (%)	98.4	98.4 / 98.4
Contamination (%)	0	0 / 0
Contigs	126	141 / 141
Largest contig (bp)	69,880	57,643 / 57,403
N50 (bp)	22,304	15,814 / 15,975
G+C content (%)	35.1 ± 1.39	35.2 ± 1.82 / 35.2 ± 1.85
Protein coding genes	2,052	1,787 / 1,806
Average contig coverage	41.56 ± 9.46	428.41 ± 127.88 / 501.76 ± 169.69

Supplementary Table 2. Key predicted gene functions encoded by the *Methanobacterium* Type I and Type II MAGs.

[Refer to Table_S2.xlsx attachment]

Supplementary Table 3. Protein affiliations, coverage profiles, and GC content values used to assign unbinned contigs encoding F_{420}-dependent methylenetetrahydromethanopterin dehydrogenase (Mtd) to Type I and Type II MAGs.

	**Genome encoding most closely related protein per BLASTp**	**Contig coverage**	**GC content (%)**	**Genome designation**
WAB188 contig encoding Mtd homolog	*Methanobacterium* sp.	61.48	38.1	WAB188 *Methanobacterium* Type I MAG
NSHQ14B contig encoding Mtd homolog	*Methanobacterium* sp.	433.47	36.4	NSHQ14B *Methanobacterium* Type II MAG
NSHQ14C contig encoding Mtd homolog	*Methanobacterium* sp.	504.88	36.5	NSHQ14C *Methanobacterium* Type II MAG

Supplementary Table 4. The distribution of F_{420}-reducing (Frh: Group 3a [NiFe]-hydrogenase), methyl viologen reducing (Mvh: Group 3c [NiFe]-hydrogenase), and multiple resistance and pH adaptation module – membrane-bound (Mrp-Mbh: Group 4 [NiFe]-hydrogenase) protein complex among members of the Methanobacteria and closely related taxa.

[Refer to Table_S4.xlsx attachment]

Supplementary Table 5. Potential rates of biological methanogenesis from formate and bicarbonate by planktonic microbial communities in well water samples collected from the Samail Ophiolite in 2020. Potential rates of biological substrate transformation were determined via microcosm assays using well waters collected from the Samail Ophiolite in 2020. The average rates of methane generation observed in four replicate abiological controls were subtracted from values in four replicate biological assays (Avg) and their combined standard deviations (SD) are presented at five timepoints over an 8-week time course series. *P*-values were determined between biological assays and abiological controls at each timepoint via Student's t-test assuming unequal variance for each condition.

	1 week	2 weeks	4 weeks	6 weeks	8 weeks
Methane generation in microcosms supplied with H_2 + formate					
WAB188 Avg (pmol CH_4 mL^{-1})	5144	9731	11472	25978	32815
SD	383	2115	2981	6354	6945
p-value	<0.01	<0.01	<0.01	<0.01	<0.01
NSHQ14 Avg (pmol CH_4 mL^{-1})	0	0	325	2411	4551
SD	-	-	195	1336	2632
p-value	-	-	0.02	0.02	0.02
Methane generation in microcosms supplied with H_2 + bicarbonate					
WAB188 Avg (pmol CH_4 mL^{-1})	3892	13624	21388	35158	47564
SD	5093	9084	7765	11331	9483
p-value	0.13	0.03	<0.01	<0.01	<0.01
NSHQ14 Avg (pmol CH_4 mL^{-1})	0	0	0	0	0
SD	-	-	-	-	-
p-value	-	-	-	-	-

Supplementary Table 6. Assembly statistics for single amplified genomes (SAGs) recovered from NSHQ14C.

[Refer to Table_S6.xlsx attachment]

Supplementary Table 7. Average nucleotide identity values (ANIs) among single amplified genomes (SAGs) and the NSHQ14C *Methanobacterium* Type II MAG.

[Refer to Table_S7.xlsx attachment]

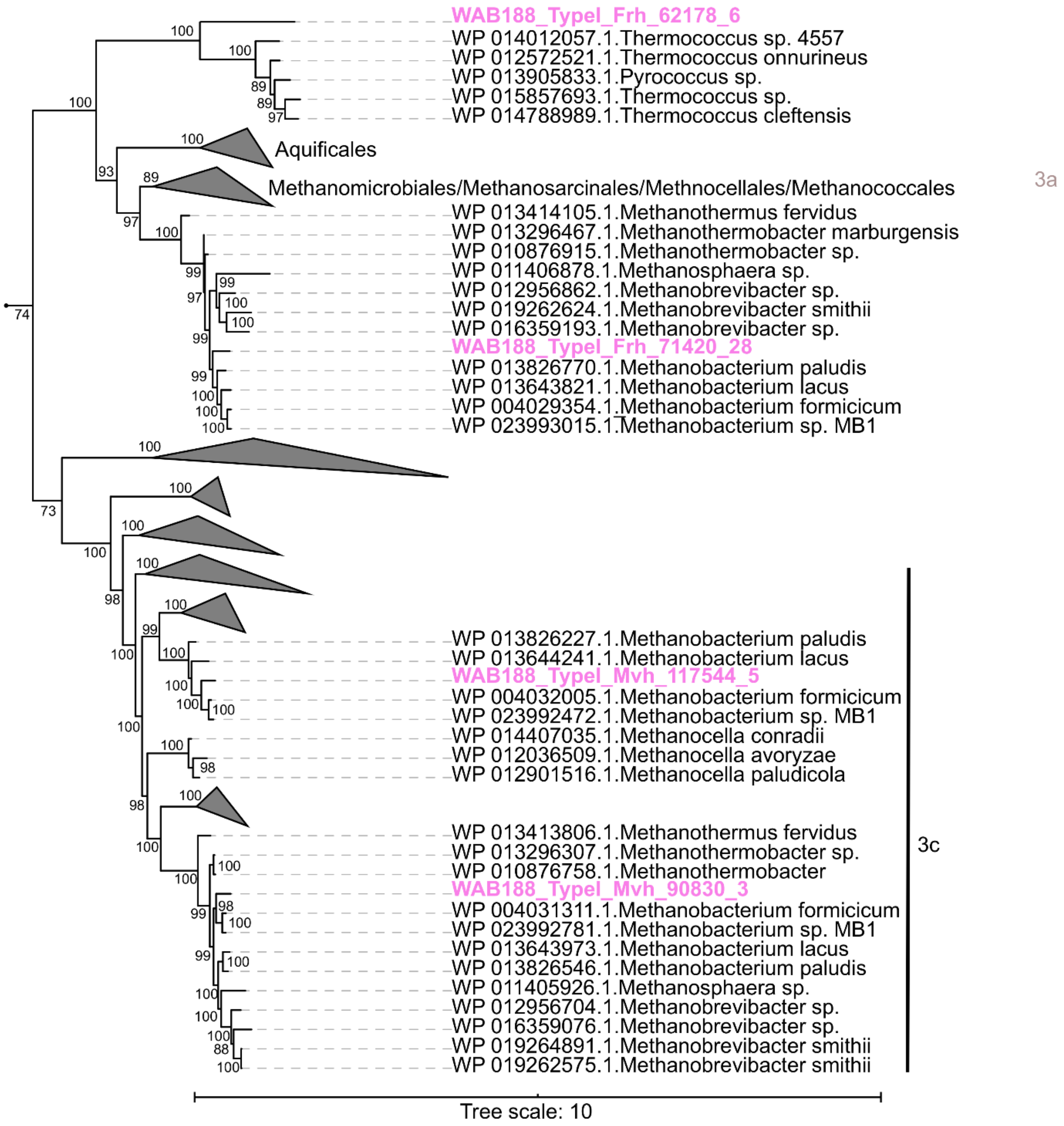

Supplementary Figure 1. Maximum Likelihood phylogenetic reconstruction of the large subunit of F_{420} (Group 3a) and methyl viologen reducing (Group 3c) [NiFe]-hydrogenases encoded in the WAB188 (Type I) MAG. Bootstraps percentages are shown for each node (out of 1,000 replicates). Clade-level triangles indicate the phylogenetic diversity within each group via side lengths that are proportional to the distances between the clade's most closely related and furthest related homologs. Branch length is relative to the scale provided for each tree. Group 3 hydrogenases were not observed in Type II genomes. Group designations follow the previous designations of Greening et al., 2016 [1].

[Refer to Figure_S2.pdf attachment]

Supplementary Figure 2. Maximum Likelihood phylogenetic reconstruction of Group 4 [NiFe]-hydrogenases, including those encoded by the WAB188 (Type I) and NSHQ14B/C (Type II) metagenome assembled genomes (MAGs). Bootstraps percentages are shown for each node (out of 1,000 replicates). Clade-level triangles indicate the phylogenetic diversity within each group via side lengths that are proportional to the distances between the clade's most closely related and furthest related homologs. Branch length is relative to the scale provided for each tree. Group designations follow the previous designations of Greening et al., 2016 [1].

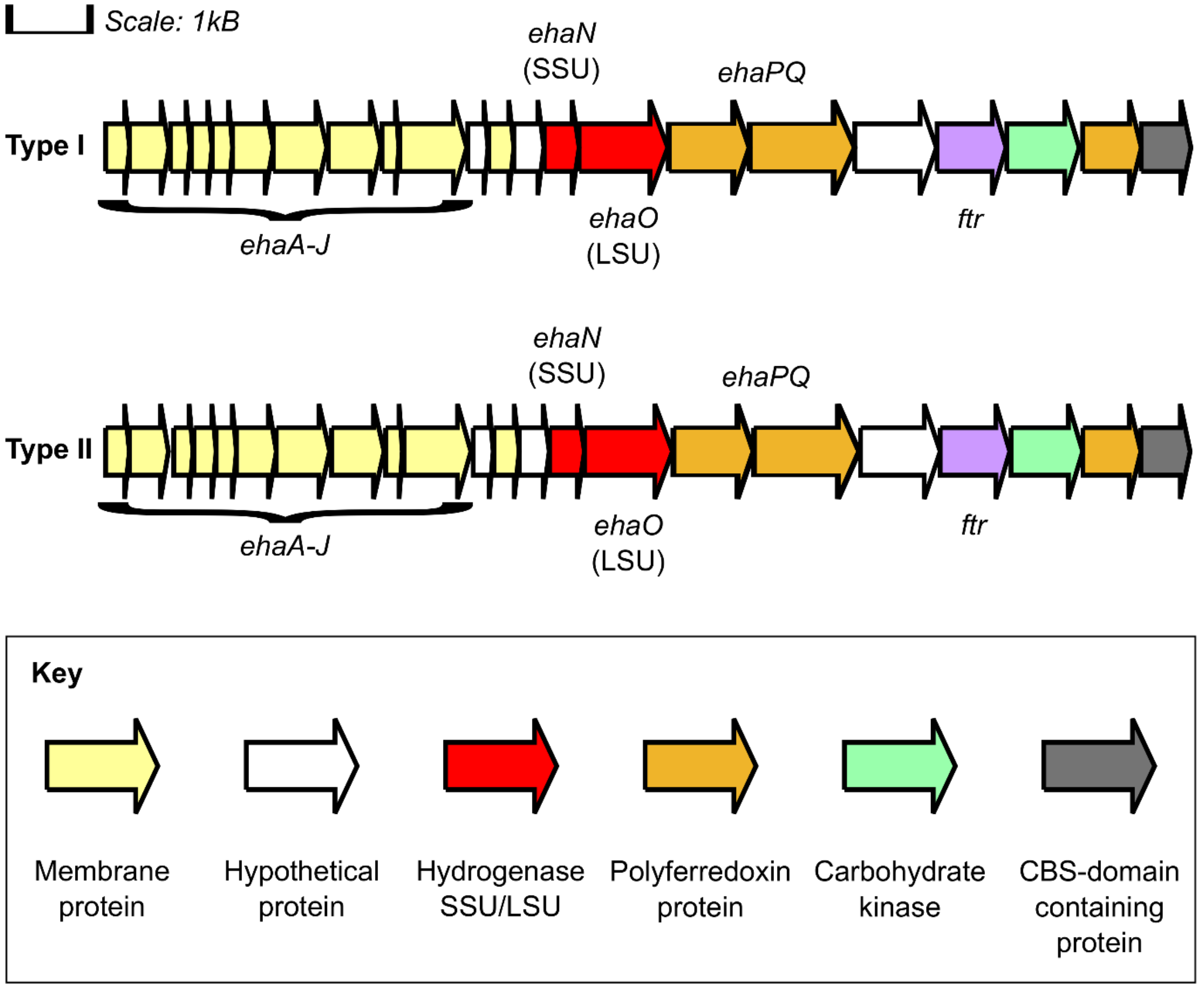

Supplementary Figure 3. Genes flanking the energy conserving Group 4 [NiFe]-hydrogenase, Eha, encoded in WAB188 (Type I) and NSHQ14B/C (Type II) metagenome assembled genomes (MAGs). Gene lengths are relative to the scale provided at the top of the figure. Gene designations are based upon CDD and BLASTp matches in addition to consensus with Tersteegen et al., 1999 [2]. Gene colors correspond to functional groupings displayed in the key at the bottom of the figure.

```
FrhB_M.marburgensis      RFLADKIKLLVGIYCMENFPYTSLQTFICEKLGVSMELVEKMDIGKGKFWVYTQDDVLT- 178
FdhB_M.formicicum        -KVNADNVIMVGLNCGGTMPPVKGRQMMEEFYEVDPDSVVKEEIAKGKLIVETEDGTEKE 174
FdhB_M.typeII            -QIEKDNVYKIGLNCGGTLMPVTARRMIDLFYEVDPDDVLKEEIDKGKFIIELKDGTHKE 169

FrhB_M.marburgensis      --LPLKETHGY-EQAGCKICKDYVAELADVSTGSVGSPD----GWSTVITRTDAGDSIFK 231
FdhB_M.formicicum        IPIDELEDEGFGRRTNCRRCEVNIPRMADLACGNWGVIGPLAGKATFIEVCSPKGAEVLE 234
FdhB_M.typeII            VKIDDLEERGYGRRSNCQRCDIMVPRNADIACGNWGAEP----GWTFIEINTEKGKELVE 225
```

Supplementary Figure 4. Multiple sequence alignment of the Group 3 F_{420}-reducing [NiFe]-hydrogenase beta subunit (FrhB) encoded by *Methanothermobacter marburgensis*, the formate dehydrogenase beta subunit (FdhB) encoded by *Methanobacterium formicicum*, and FdhB encoded in the *Methanobacterium* NSHQ14C Type II metagenome assembled genomes (MAGs). Black text represents amino acid residues involved in F_{420} coordination in FrhB [3] and corresponding residues in FdhB proteins.

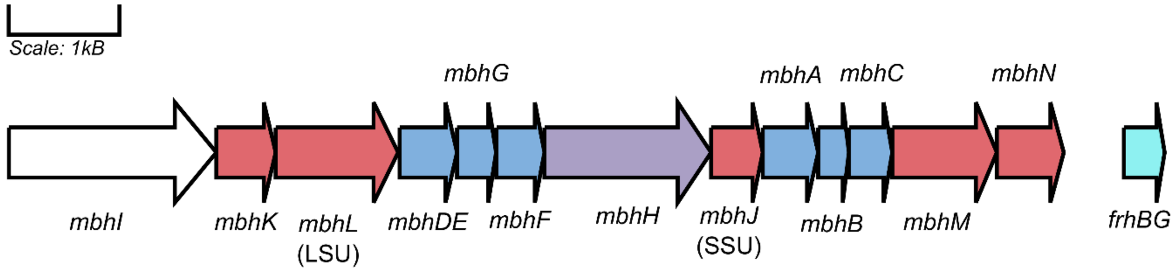

Supplementary Figure 5. Genes flanking the multiple resistance and pH adaptation - membrane bound hydrogenase protein complex (Mrp-Mbh) energy converting (Group 4) [NiFe]-hydrogenases encoded in Type II metagenome assembled genomes (MAGs). The color scheme is based on functional designations outlined by Schut et al., 2012 [4]. Red arrows represent genes encoding subunits of the [NiFe]-hydrogenase (Mbh) involved in reversible hydrogen oxidation, blue arrows represent genes putatively involved in ion transport (Mrp), the purple arrow represents *mbhH*, which is putatively a component of both Mbh and Mrp, the white arrow represents *mbhI* which is unique to the Mrp-Mbh complex enzyme class (i.e. not encoded in Mrp or Mbh alone), and the cyan arrow represents the fused homolog of Group 3 F_{420}-reducing [NiFe]-hydrogenase FrhB and FrhG subunits. Gene lengths are relative to the scale provided at the top of the figure.

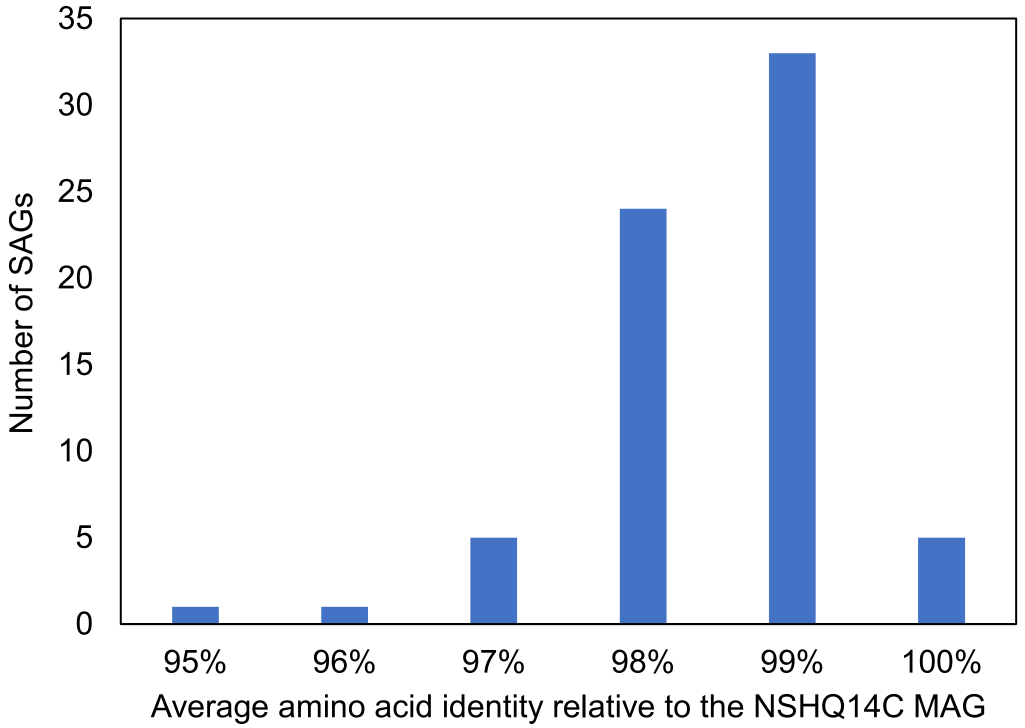

Supplementary Figure 6. Average amino acid identity (AAI) between single amplified genomes (SAGs) and the NSHQ14C (Type II) metagenome assembled genome (MAG). Amino acid identities of protein homologs shared by SAGs and the NSHQ14C Type II MAG were averaged to arrive at an average AAI for each SAG relative to the NSHQ14C Type II MAG.

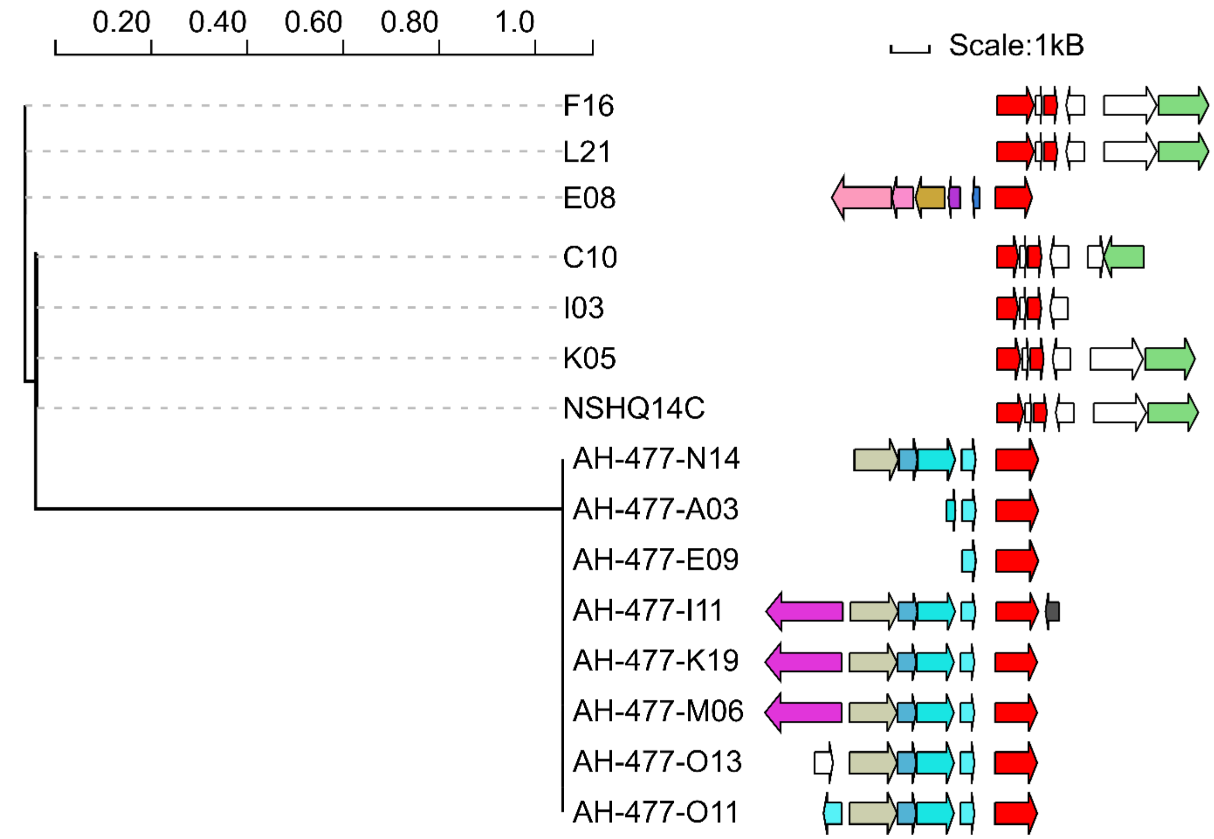

Supplementary Figure 7. Phylogenetic reconstruction of C39 peptidase protein orthologs and their genome arrangements in single amplified genomes (SAGs) and the NSHQ14C Type II metagenome assembled genome (MAG). Branch length is relative to the scale provided at the top of the figure indicating average substitutions per site. The proximal 5 ORFs upstream and downstream of the C39 peptidase are depicted to the right of the terminals. Red arrows represent genes encoding C39 peptidase orthologs, white arrows represent genes encoding hypothetical proteins, black arrows represent genes encoding transposase proteins, and the remaining colored arrows represent genes encoding other functional proteins. Gene lengths are relative to the scale provided at the top of the figure.

References.

1. Greening, C., Biswas, A., Carere, C.R., Jackson, C.J., Taylor, M.C., Stott, M.B. *et al.* Genomic and metagenomic surveys of hydrogenase distribution indicate H_2 is a widely utilised energy source for microbial growth and survival. ISME J. 2016; 10: 761-777.
2. Tersteegen, A. and Hedderich, R. Methanobacterium thermoautotrophicum encodes two multisubunit membrane-bound [NiFe] hydrogenases: Transcription of the operons and sequence analysis of the deduced proteins. Eur. J. Biochem. 1999; 264: 930-943.
3. Mills, D.J., Vitt, S., Strauss, M., Shima, S., and Vonck, J. De novo modeling of the F_{420}-reducing [NiFe]-hydrogenase from a methanogenic archaeon by cryo-electron microscopy. Elife 2013; 2: e00218.
4. Schut, G.J., Boyd, E.S., Peters, J.W., and Adams, M.W.W. The modular respiratory complexes involved in hydrogen and sulfur metabolism by heterotrophic hyperthermophilic archaea and their evolutionary implications. FEMS Microbiol. Rev. 2013; 37: 182-203.
