# Supplementary figures and images for: Diversification of methanogens into hyperalkaline serpentinizing environments through adaptations to minimize oxidant limitation
Source: ISME J. 2020 Nov 30;15(4):1121–35. doi: 10.1038/s41396-020-00838-1 (PMC8115248; doi:10.1038/s41396-020-00838-1)

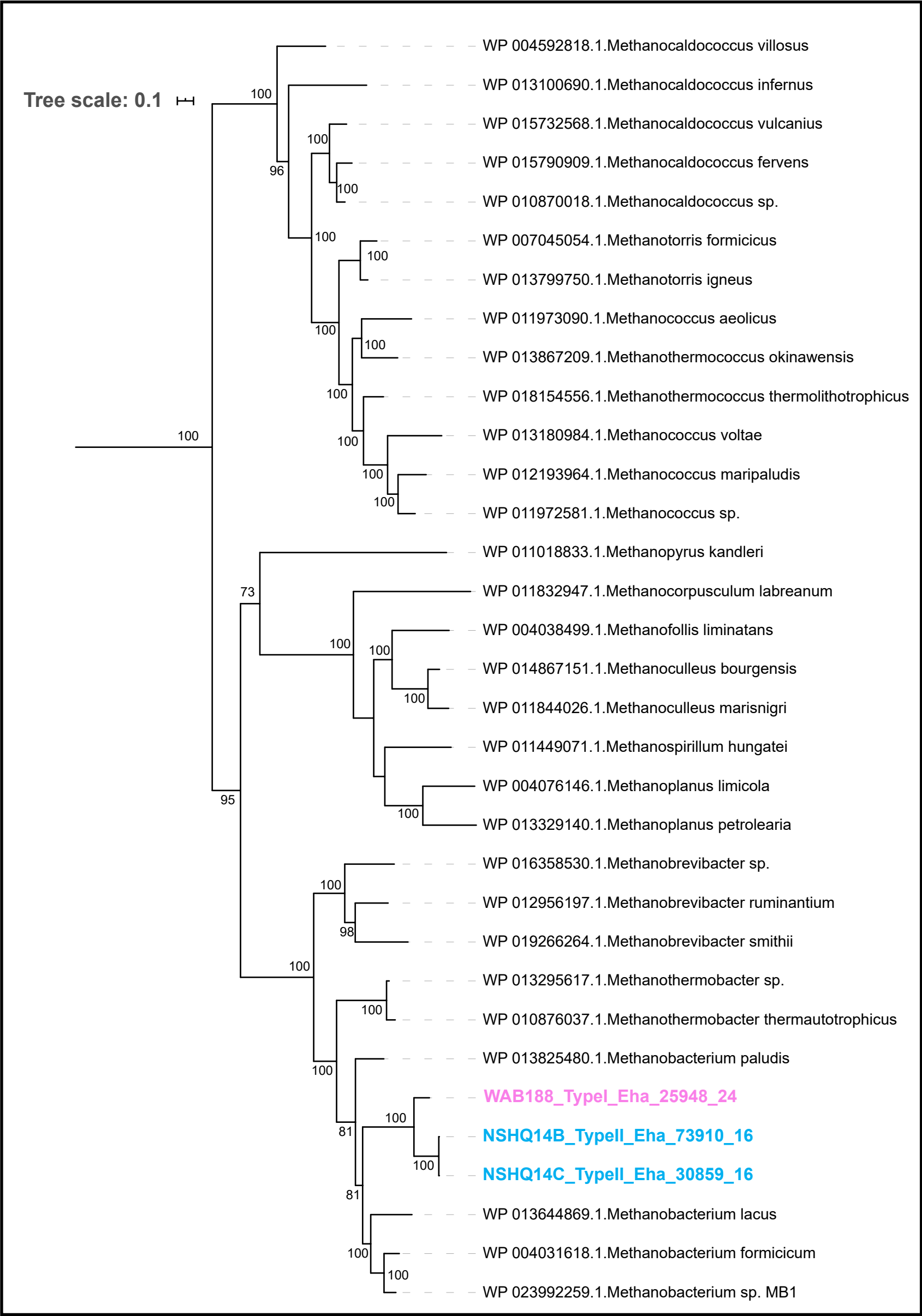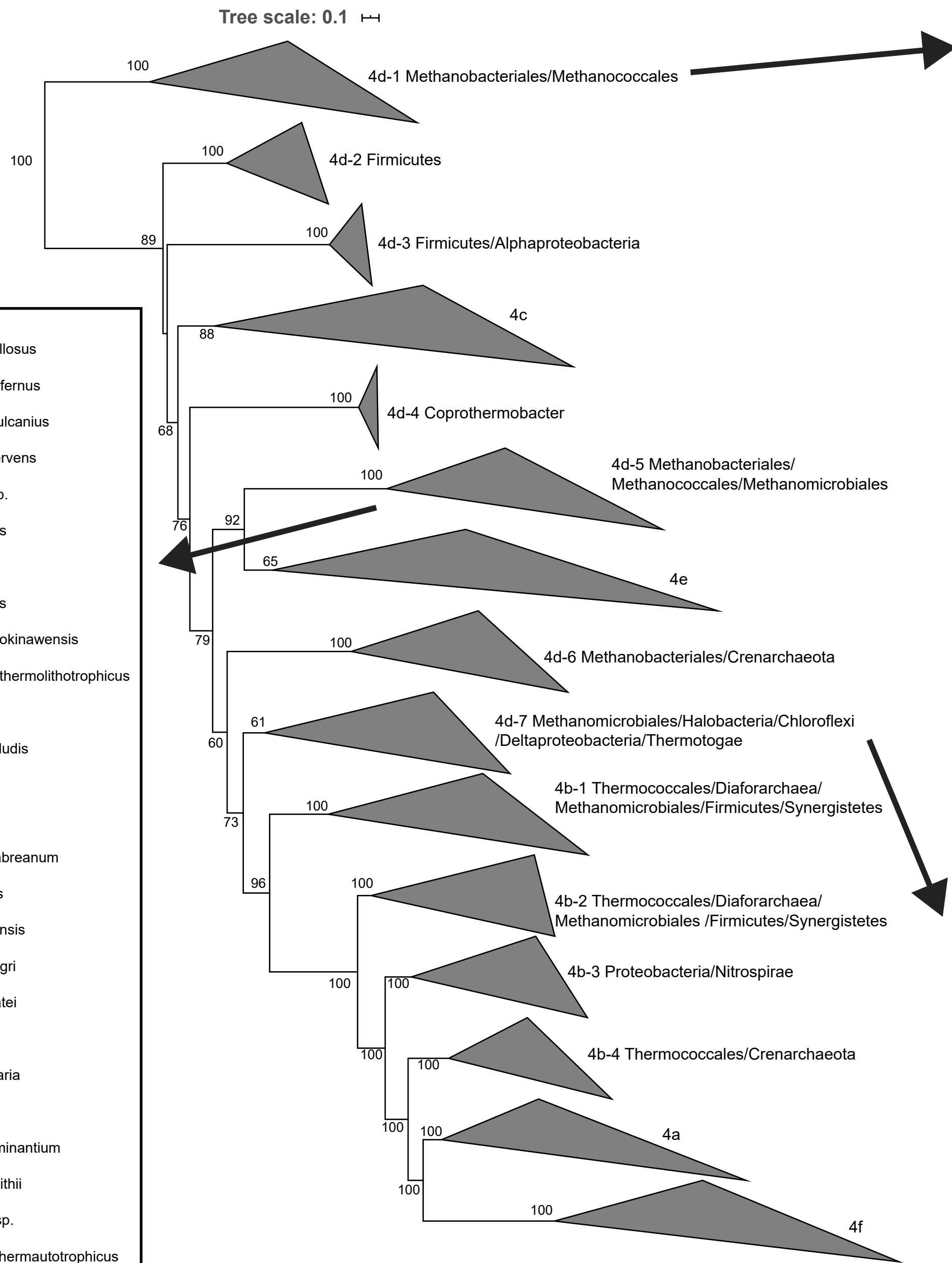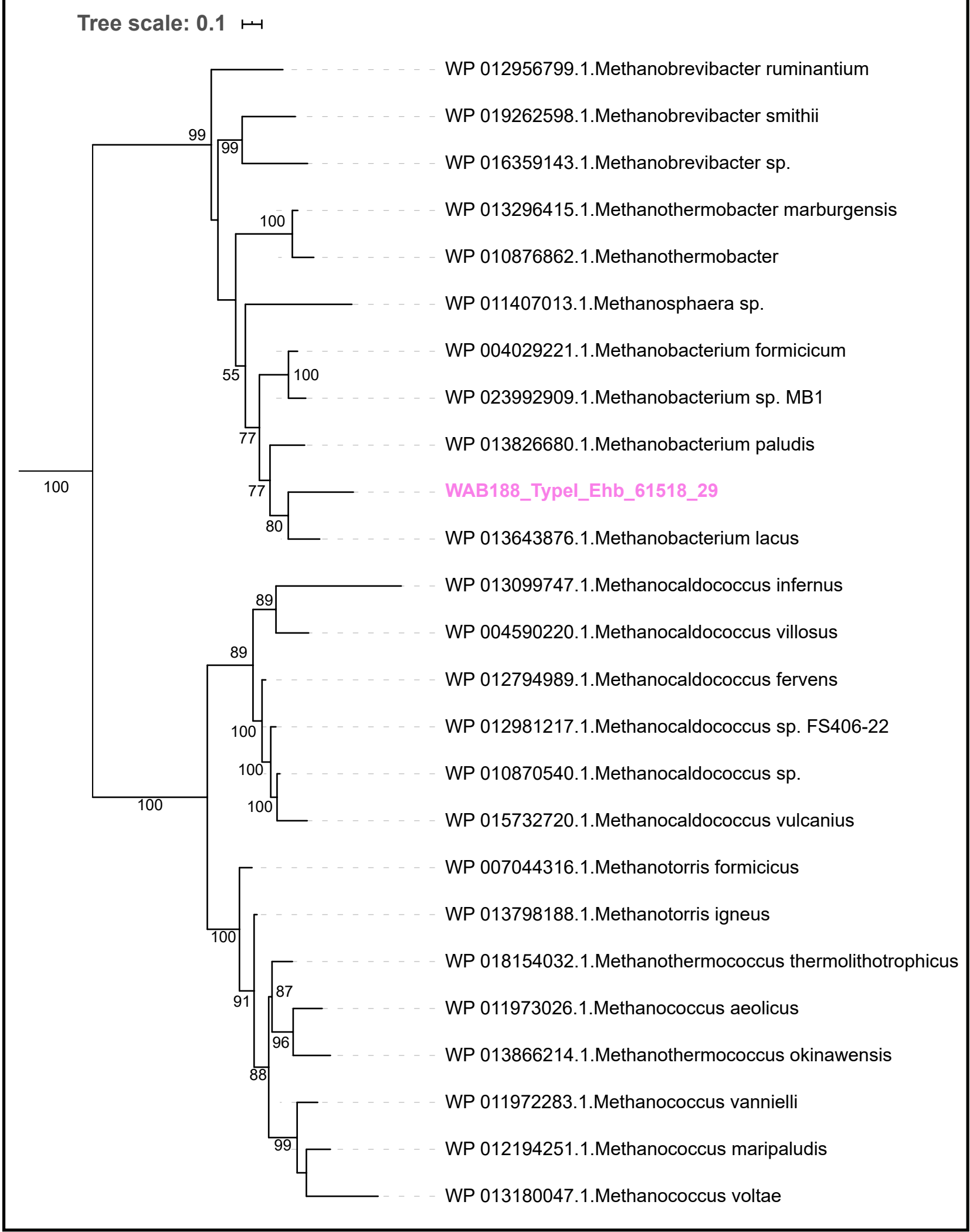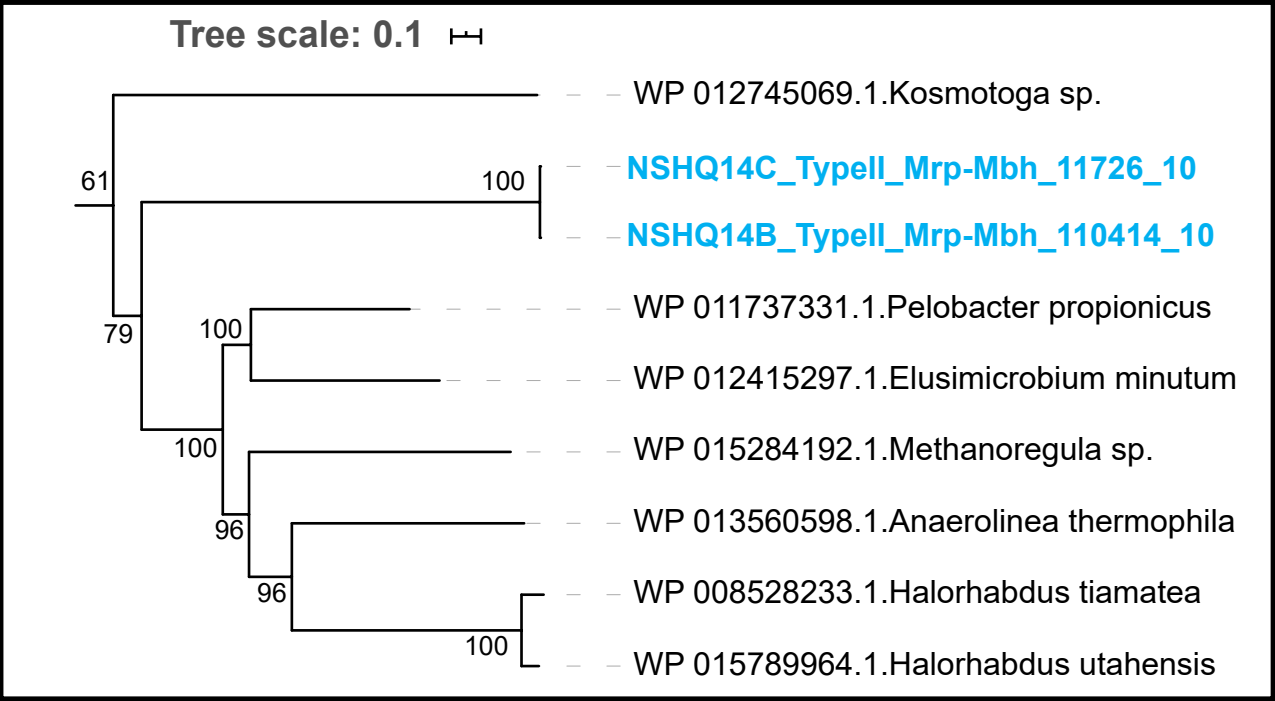

Supplement: Supplementary file 2 — Supplemental Figure 2 [file 41396_2020_838_MOESM2_ESM.pdf]
